# Every‐other‐day palonosetron plus aprepitant for prevention of emesis following induction chemotherapy for acute myeloid leukemia: A randomized, controlled study from the “Rete Ematologica Pugliese”

**DOI:** 10.1002/cam4.2628

**Published:** 2019-11-14

**Authors:** Nicola Di Renzo, Lorella Melillo, Fernando Porretto, Michela Dargenio, Vincenzo Pavone, Domenico Pastore, Patrizio Mazza, Donato Mannina, Anxur Merenda, Nicola Cascavilla, Giuseppina Greco, Rosella Matera, Erminio Bonizzoni, Luigi Celio, Maurizio Musso

**Affiliations:** ^1^ Department of Hematology and Stem Cell Transplant Presidio Ospedaliero Vito Fazzi Lecce Italy; ^2^ Hematology Unit IRCCS Casa Sollievo della Sofferenza San Giovanni Rotondo Italy; ^3^ Hematology Unit Casa di Cura La Maddalena Palermo Italy; ^4^ Hematology Unit Ospedale Cardinale G. Panico Tricase Italy; ^5^ Hematology Unit Ospedale A. Perrino Brindisi Italy; ^6^ Hematology Unit Ospedale S.G. Moscati Taranto Italy; ^7^ Hematology Unit A.O. Ospedali Riuniti Papardo‐Piemonte Messina Italy; ^8^ Hematology Unit Ospeale Civico Palermo Italy; ^9^ Section of Medical Statistics, Biometry and Epidemiology University of Milan Milan Italy; ^10^ Department of Medical Oncology and Hematology Fondazione IRCCS Istituto Nazionale dei Tumori Milan Italy

**Keywords:** acute myeloid leukemia, AML, aprepitant, CINV, emesis, nausea, palonosetron

## Abstract

**Background:**

Compared with older 5‐HT_3_ receptor antagonists, palonosetron requires fewer drug administrations to prevent chemotherapy‐induced nausea and vomiting (CINV) following multiple‐day chemotherapy. We conducted a phase II multicenter study comparing palonosetron plus aprepitant to palonosetron alone in patients undergoing a range of induction chemotherapy regimens for acute myeloid leukemia (AML).

**Methods:**

Patients were randomized to palonosetron (0.25 mg) every other day until the last dose of chemotherapy alone or with aprepitant on days 1‐3. Patients mainly received an anthracycline on days 1‐3 plus cytarabine administered for 5‐10 days. The primary end point was complete response (CR; no emesis and no rescue medication) over the whole study period (days of chemotherapy plus two additional days). Unplanned analysis of time to anti‐emetic treatment failure (TTF) was also performed.

**Results:**

Of the 134 patients enrolled in the study, 130 were evaluable: 68 subjects received palonosetron plus aprepitant and 62 received palonosetron alone. Although the primary end point of CR was similar between the treatment arms (72% vs 69%; *P* = .55), a higher proportion of patients treated with palonosetron plus aprepitant were free from nausea during the whole study period (43% vs 27%; *P* = .03). There was also a significant difference in favor of the two‐drug regimens in TTF (median: 5 days vs 3 days; *P* = .03).

**Conclusions:**

The study suggests that every‐other‐day palonosetron plus 3‐day aprepitant can add clinical benefit to the control of CINV caused by multiple‐day, corticosteroid‐free chemotherapy for AML. In this challenging setting of CINV, further investigations of palonosetron in combination with aprepitant administered with an expanded schedule are warranted.

http://ClinicalTrial.gov identifier: NCT02205164.

## Graphical abstract

In this randomized study on acute myeloid leukemia patients treated with multiple‐day induction chemotherapy, the authors investigated the efficacy of a simplified antiemetic prophylaxis consisting of every‐other‐day palonosetron plus 3‐day aprepitant compared to palonosetron alone. Although similar rates of complete response were observed, the combination regimen significantly improved control of nausea in this challenging setting of chemotherapy‐induced nausea and vomiting.

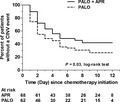

## 1 INTRODUCTION

Chemotherapy‐induced nausea and vomiting (CINV) is a common and debilitating side effect associated with anticancer chemotherapy.^1^ For young adults and fit elderly patients with acute myeloid leukemia (AML), cytarabine with an anthracycline remains the mainstay of induction chemotherapy.^2^ Anthracyclines as well as cytarabine (>1000 mg/m^2^ per day) carry a moderate emetic risk as single agents (incidence of acute emesis without antiemetics, 30%‐90%) but their combination may substantially increase the emetic risk.^3^, ^4^ The emetogenic potential of these regimens is further increased by the schedule of drug administration, with overlapping acute and delayed CINV triggered by each agent over multiple days. Therefore, patients are at risk of CINV throughout the entire treatment period, which makes the development of an effective antiemetic coverage for each day challenging.^5^ Also, the use of corticosteroids for preventing CINV in patients with hematologic malignancies must be avoid, as they may further worsen immune response in these already severely immunesuppressed subjects.^6^

Palonosetron, a second‐generation 5‐HT_3_ receptor antagonist (5‐HT_3_RA), has a markedly superior receptor binding affinity and plasma half‐life compared to older antagonists.^7^ Therefore, if compared with first‐generation 5‐HT_3_RA, fewer palonosetron administrations are needed to obtain at least the same degree of protection against CINV caused by multiple‐day chemotherapy.^4^, ^8^, ^9^ In addition, randomized studies showed that palonosetron plus single‐dose dexamethasone may allow to reduce patient exposure to steroids without compromising the overall antiemetic outcome over 5 days on the high emetogenic risk combination of the anthracycline plus cyclophosphamide regimen as well as other moderately emetogenic treatments.^10^, ^11^

A multicenter, observational study including 77 patients with AML who received moderately to highly emetogenic chemotherapy (HEC) showed that a first‐generation 5‐HT_3_RA alone induced complete response (CR) only in 47% of patients during the whole 5‐day observation period.^6^ A suboptimal control of symptoms occurred mainly in the observation delayed period (ie, 24 hours after the start of chemotherapy). In addition, a randomized study showed that more than 50% of the patients with AML receiving multiple doses of palonosetron still required rescue medication.^9^

The neurokinin‐1 receptor antagonist (NK‐1RA) aprepitant, the first agent in the class, was shown to cause an additive effect when combined with a 5‐HT_3_RA and corticosteroids for the prevention of acute and delayed CINV in patients receiving single‐day chemotherapy regimens.^12^ Therefore, we conducted an investigator‐initiated, multicenter, open‐label, randomized, phase II trial to compare every‐other‐day palonosetron in combination with aprepitant, at the approved 3‐day oral schedule, versus palonosetron alone for CINV prevention during the entire course of therapy in AML patients undergoing a range of induction chemotherapy regimens.

## 2 PATIENTS AND METHODS

### 2.1 Study design

This study was a multicenter, randomized, open‐label, phase 2 trial. The study was conducted in accordance with the Good Clinical Practice guidelines, in nine Italian centers between December 2011 and July 2014, after approval by the local institutional review board. All patients filled a written informed consent form before being registered in the study. A computer‐generated randomization list was used to allocate treatment on a one‐to‐one basis for the two study arms. This task was conducted at the Coordination Centre (Associazione Salentina “Angela Serra – Italia Memmi Ferrari”; Lecce, Italy) independent of all other trial procedures. Patients with AML receiving multiple‐day induction chemotherapy were eligible.

For all patients, antiemetic coverage consisted of a single intravenous dose of palonosetron (0.25 mg) given 30 minutes prior to the administration of chemotherapy on day 1. In Italy as well as other Western countries, the standard dose of palonosetron is 0.25 mg intravenously per day. Additional palonosetron doses were administered every other day and the total number of doses of palonosetron was according to the chemotherapy schedule. In addition, patients were randomly assigned to receive either 125 mg aprepitant (po) on day 1 and 80 mg once per day on days 2 and 3, or no additional antiemetic besides palonosetron. No corticosteroids were permitted during the study as antiemetic prophylaxis. All patients were required to discontinue any drug with potential antiemetic effects before the study. Rescue antiemetics were given as needed, although additional palonosetron or aprepitant was not permitted. Concomitant medications and therapies deemed necessary for supportive care were allowed (ie, antibiotics and antifungal prophylaxis).

The primary objective of the study was to demonstrate the superiority of palonosetron plus aprepitant over palonosetron alone based on the proportion of patients with CR during the entire study period (days of chemotherapy administration plus 2 days after chemotherapy completion), defined as no emesis with no use of rescue medication. Secondary end points included complete control (CC; defined as CR, and no more than mild nausea), no vomiting, no nausea, and no use of rescue medication in the acute period (same days of chemotherapy administration) or during the entire study period. Thus, the observation period depended on the duration of the given chemotherapy regimen. In an unplanned analysis, all efficacy end points were also evaluated on aprepitant dosing days.

Serious unexpected adverse events (AEs) were also recorded during the study. Any serious AE judged by the investigator to be possibly, probably, or definitely related to palonosetron and/or aprepitant was graded according to the common terminology criteria for AEs, version 3.0.

### 2.2 Patients

Patients were included if aged 18 years or older and diagnosed with AML or high‐risk myelodysplastic syndrome (according to the International Prognostic Scoring System) and eligible to receive induction chemotherapy with the 3 + 7 regimen (daunorubicin plus cytarabine) or a fludarabine‐based regimen (fludarabine plus cytarabine with or without an anthracycline). Patient eligibility was subjected to absence of nausea and vomiting at baseline. Adequate hepatic (aspartate transaminase [AST] and alanine transaminase [ALT] ≤2 times the upper normal limit) and renal (creatinine ≤1.5 times the upper normal limit) functions were required. Patients were excluded from the study if they had known hypersensitivity to study medications. Other exclusion criteria included active infection requiring intravenous antibiotics, myocardial infarction within 6 months before the study, and psychiatric disorders interfering with ability to comply with study protocol.

### 2.3 Assessments

All study subjects were inpatients for the duration of the study and were directly observed by trained nurses for episodes of CINV. Patients were asked to complete a standard diary on a daily basis for the whole observation period. Patients recorded daily any episodes of emesis or nausea. The daily use of rescue therapy, defined as any medication taken to treat established nausea or emesis, was recorded by nurses. Nausea was graded daily using a four‐point scale (no nausea, mild, moderate, or severe). Data about the patient's quality of life were not collected in this study.

### 2.4 Statistical analysis

The sample size (N = 120) was calculated with respect to a two‐sided *Z* test with pooled variance of the primary end point, the proportion of CR in the whole study period. On the basis of the available evidence when the study was designed, a difference of at least 25% (35% vs 60%) was determined to cause a significant effect (at 0.05 level), with a power of 0.80.^9^ Assuming a 10% drop‐out rate, 134 patients needed to be enrolled. Primary and secondary efficacy end points were evaluated using an intention‐to‐treat approach, which included all patients given study medication and chemotherapy with at least one efficacy assessment within the observation study period. All analyses of the efficacy end points were based on logistic regression models, including treatment, gender, age, and chemotherapy duration as explanatory variables. Results were reported as odds ratios with associated 95% confidence interval and two‐sided *P*‐values. The Fisher's exact test was used to assess the association between treatment and response on each study day. Only the primary analysis (CR in the whole period) was controlled for type I error; therefore, analyses of all other end points should be viewed as exploratory and no adjustments were made for multiplicity. A statistical significance level of .05 was used to indicate a difference between the treatment groups.

A separate analysis of total control of CINV evaluated the probability that a patient would remain free from CINV events (ie, emesis and/or use of rescue medication and/or nausea) over the entire study period. This unplanned analysis was performed by using the Kaplan‐Meier method, and any patient who experienced CINV was considered as treatment failure. Treatment arms were compared using log‐rank test.

## 3 RESULTS

### 3.1 Patient characteristics

**Figure 1:**
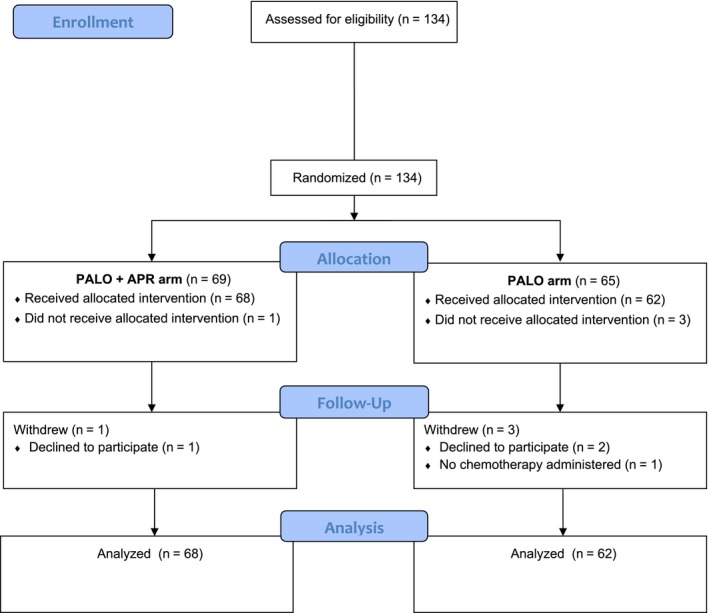
CONSORT flow diagram. Abbreviations: APR, aprepitant; PALO, palonosetron

Among the total of 134 consecutive patients enrolled in the study, 130 were fully evaluated: 68 in the palonosetron plus aprepitant arm and 62 in the palonosetron alone arm (Figure 1). Patient characteristics and the type of chemotherapy administered were similar between the two treatment arms (Table 1). The median duration of chemotherapy administration was the same in both arms (7 days; min‐max: 5‐10 days). No patient in either arm received concurrent pain medications or intravenous antibiotics during the study period.

**Table 1:** Baseline patient characteristics

Characteristic	PALO plus APR N (%)	PALO alone N (%)	*P*
No. of patients	68	62	
Age, y			
Median (min‐max)	56 (19‐78)	60.5 (20‐81)	.70^d^
Age ≤60 y	44 (64.7)	31 (50.0)	.09^e^
Female sex	30 (44.1)	30 (48.4)	.62^e^
Diagnosis of AML	65 (95.6)	61 (98.4)	.35^e^
ECOG performance status			
0‐1	63 (92.6)	55 (88.7)	.46^e^
2	5 (7.4)	5 (8.1)	
Unknown	0	2 (3.2)	
No alcohol consumption	56 (82.4)	54 (87.1)	.45^e^
Presence of symptoms at start	17 (25)	16 (25.8)	.16^e^
Type of chemotherapy			.27^e^
Daunorubicin + low‐dose cytarabine^a^	56 (82.4)	42 (67.7)	
Cytarabine + fludarabine + liposomal doxorubicin^b^	3 (4.4)	4 (6.5)	
Cytarabine + fludarabine + idarubicin^b^	6 (8.8)	10 (16.1)	
Cytarabine + fludarabine^b^	3 (4.4)	6 (9.7)	
Anthracycline dosing for multiple days^c^	62 (91.2)	52 (83.4)	.20^e^
Chemotherapy duration			.12^e^
5 d	8 (11.8)	15 (24.2)	
7 d	27 (39.7)	25 (40.3)	
10 d	33 (48.5)	22 (35.5)	

Abbreviations: AML, acute myeloid leukemia; APR, aprepitant; ECOG, eastern cooperative oncology group; PALO, palonosetron.

a Cytarabine dose of 100‐200 mg/m^2^ per day.

b Cytarabine dose > 1000 mg/m^2^ per day.

c Daunorubicin or idarubicin.

d Unpaired *t* test (two‐sided).

e Chi‐square test (two‐sided).

### 3.2 Efficacy

Efficacy results are shown in Table 2. The primary analysis of CR showed no significant difference between the two treatment arms during the whole study period (72% vs 69%; *P* = .55). Also, no statistically significant differences were found between the treatment arms for any other predefined end point of efficacy, except for nausea. During the whole period, significantly more patients receiving the combination of palonosetron and aprepitant than those receiving palonosetron alone were free from nausea (43% vs 27%; *P* = .03). Moreover, a trend emerged in favor of patients who received the two‐drug regimen and reported no nausea during the acute period (43% vs 29%; *P* = .05).

**Table 2:** Efficacy results in each observation period by treatment

End point by study period^a^	PALO plus APR n/N (%)	PALO alone n/N (%)	OR (95% CI)	*P*
Complete response				
Whole^b^	49/68 (72.1)	43/62 (69.4)	1.27 (0.57, 2.81)^c^	.55
Acute	53/68 (77.9)	45/62 (72.6)	1.52 (0.65, 3.55)^c^	.33
Days 1 to 3	64/68 (94.1)	49/62 (79.0)	4.95 (1.43, 17.1)^d^	.01
Complete control				
Whole	40/68 (58.8)	35/62 (56.5)	1.34 (0.63, 2.87)^c^	.44
Acute	43/68 (63.2)	36/62 (58.1)	1.55 (0.72, 3.37)^c^	.26
Days 1 to 3	58/68 (85.3)	45/62 (72.6)	2.33 (0.95, 5.72)^d^	.06
No emesis				
Whole	29/68 (42.6)	18/62 (29.0)	1.68 (0.72, 3.89)^c^	.22
Acute	29/58 (42.6)	18/62 (29.0)	2.29 (0.93, 5.65)^c^	.07
Days 1 to 3	64/68 (94.1)	49/62 (79.0)	3.78 (1.20, 11.9)^d^	.02
No nausea				
Whole	29/68 (42.6)	17/62 (27.4)	2.40 (1.08, 5.35)^c^	.03
Acute	29/68 (42.6)	18/62 (29.0)	2.21 (0.99, 4.90)^c^	.05
Days 1 to 3	43/68 (63.2)	31/62 (50.0)	1.79 (0.87, 3.68)^d^	.11
No rescue medication				
Whole	54/68 (79.4)	47/62 (75.8)	1.30 (0.55, 3.08)^c^	.54
Acute	57/68 (83.8)	49/62 (79.0)	1.51 (0.59, 3.86)^c^	.39
Days 1 to 3	66/68 (97.1)	54/62 (87.1)	5.48 (1.07, 28.1)^d^	.04

Abbreviations: APR, aprepitant; CI, confidence interval; n, number of responding patients; N, number of patients; OR, odds ratio; PALO, palonosetron.

a Whole period: up to 48 hours after the end of chemotherapy; acute period: within days of chemotherapy administration; days 1‐3: within 3 days after chemotherapy initiation.

b Primary efficacy end point.

c Based on logistic regression models including treatment, gender, age, and chemotherapy duration as explanatory variables.

d Based on logistic regression models including treatment, gender and age.

### 3.3 Unplanned efficacy analyses

**Figure 2:**
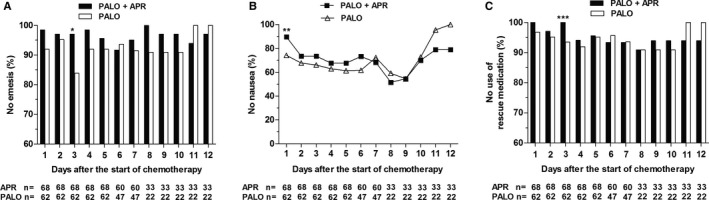
Proportions of emesis‐free patients (A), nausea‐free patients (B), or patients with no use of rescue medication (C) at different days of the study for each treatment arm. Due to the different chemotherapy regimens used, the sample size decreases in time. Abbreviations: APR, aprepitant; PALO, palonosetron. *P = .01 vs the palonosetron alone arm (two‐sided Fisher's exact test). **P = .02 vs the palonosetron alone arm. ***P = .04 vs the palonosetron alone arm

Proportions of emesis‐free patients on each day are shown in Figure 2A; the daily assessment of emesis showed a statistically significant difference on day 3 (*P* = .01). No statistically significant differences between the study arms related to no‐emesis patients were observed on days 4 to 12. On day 1, 90% of patients in the palonosetron plus aprepitant arm were free from nausea as compared with 74% in the palonosetron alone arm (*P* = .02; Figure 2B). On days 2 to 6, more patients receiving aprepitant reported no nausea compared to patients in the palonosetron alone arm, but statistical test failed to prove significant differences between the treatment arms. The proportion of patients not using rescue antiemetics was similar between the treatment arms during the entire study, with the exception of day 3 (Figure 2C; 100% the combination vs 94% palonosetron alone; *P* = .04). A significantly higher proportion of patients experiencing CR, no emesis, and no use of rescue medication was found in the palonosetron plus aprepitant arm, as compared to the palonosetron alone arm, by the end of day 3 after chemotherapy initiation (Table 2). Although no significant difference between the arms was observed in nausea control on aprepitant dosing days, a trend was present in favor of a higher rate of CC in the combination arm over the first 3 days (85% vs 73%; *P* = .06).

**Figure 3:**
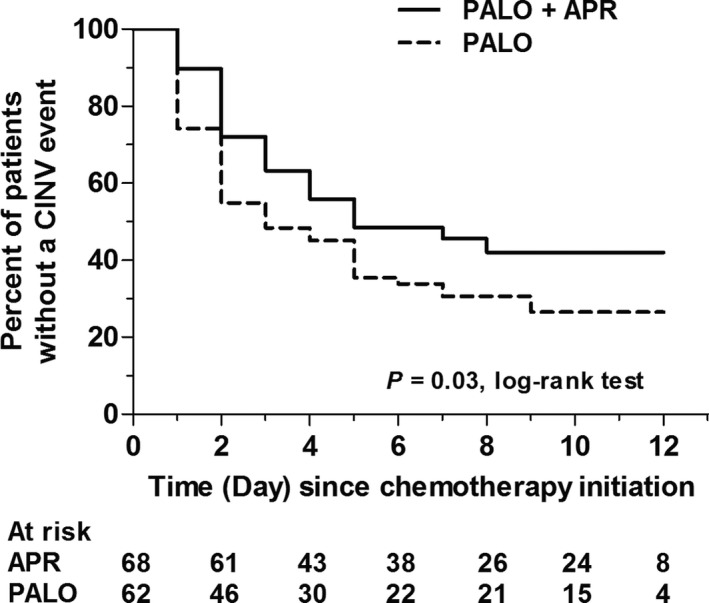
Kaplan‐Meier analysis of patients with no CINV events over the whole study period. Any patient who experienced a CINV event (ie, emesis and/or rescue medication use and/or mild‐to‐severe nausea) was considered as treatment failure. Abbreviations: APR, aprepitant; CINV, chemotherapy‐induced nausea and vomiting; PALO, palonosetron

The percentage of CINV‐free patients over the whole study period was significantly greater for the combination than palonosetron alone (42% vs 27%; *P* = .03, based on log‐rank test; Figure 3). Median time to the first CINV event (ie, treatment failure) was in favor of the combination (5 vs 3 days).

### 3.4 Safety

The safety of study treatments was assessed during the entire observation period. No patient discontinued treatment due to AEs. The most common AEs considered possibly or probably related to the antiemetic agents were headache (4% vs 10%, in the experimental and control arms, respectively; *P* = .30), constipation (4% vs 3%; *P* = 1.0), and anorexia (9% vs 3%; *P* = .27).

## 4 DISCUSSION

Chemotherapy‐induced nausea and vomiting control remains a challenge in the setting of hematologic malignancies as few controlled studies have addressed this issue.^4^ To the best of our knowledge, this is the first randomized study evaluating the efficacy and safety of every‐other‐day palonosetron in combination with aprepitant in patients treated with a range of corticosteroid‐free, induction regimens for AML. In this clinical setting, we were interested in evaluating whether a more intensive prophylaxis regimen at the onset of emetogenic, multiple‐day chemotherapy could ameliorate control of CINV within the entire course of therapy. It should be noted that the rate of CR during the whole study period was higher than expected in the patients randomized to the control arm, and this may have contributed to the negative results of the primary efficacy end point. In spite of this study failed to meet the primary goal, results on secondary end points would suggest that the addition of aprepitant to palonosetron is beneficial in this setting. It must be pointed out that, in symptom control trials, outcome measures are by nature multidimensional, and the relative importance of the primary vs secondary end points can be similar. The composite end point of CR that represents the standard indicator of antiemetic efficacy, does not truly account for nausea control.^13^ Since not all patients having some degree of nausea take rescue medications, using “no use of rescue medication” as part of the CR serves as a surrogate marker for no nausea or only mild nausea. Therefore, we think that there are three principal reasons to make the findings of this study clinically relevant. First, patients only received aprepitant for 3 days as per the approved administration schedule because we were not able to implement a longer duration of aprepitant due to budget restrictions in this investigator‐initiated trial. In light of this, in an unplanned analysis the efficacy results over aprepitant dosing days indicate that the rates of patients with a CR as well as no emesis and no use of rescue medication were significantly greater in the palonosetron plus aprepitant arm compared to palonosetron alone. These results are in accordance with the literature demonstrating that aprepitant added to a 5‐HT_3_RA plus dexamethasone is able to significantly increase protection against delayed emesis in patients receiving single‐day HEC.^1^ Interestingly, in a recent retrospective study, palonosetron was superior to granisetron, when both were given alone, for the prevention of CINV in AML patients receiving chemotherapy regimens administered for more than five consecutive days.^14^ Also, palonosetron plus 3‐day aprepitant instead of granisetron plus aprepitant was found to be the optimal regimen for achieving a CC of CINV in this cohort. In the setting of multiple‐day chemotherapy, the emetogenic potential is further increased by the schedule of administration, with overlapping acute and delayed CINV caused by different chemotherapy agents over multiple days. Since 88% of the patients in this study received an anthracycline administered on days 1‐3, it is likely that the addition of aprepitant was able to ameliorate control of overlapping acute and delayed emesis caused by the moderately emetogenic anthracycline during the first 3 days.^1^, ^3^ In absolute terms, there was a 15% benefit (94% vs 79%) in both CR and CC of emesis as well as 10% (97% vs 87%) in the proportion of patients not requiring rescue medications over the first 3 days. Interestingly, in a recent, single‐arm phase II trial, when combined with an older 5‐HT_3_RA, 5‐day aprepitant was found to achieve a CC of vomiting in 74% of 38 patients undergoing 3 + 7 induction chemotherapy for AML over aprepitant dosing days.^15^

Second, although the effect of aprepitant on nausea control was less apparent during the first 3 days, a trend emerged toward a higher proportion of patients with a CC, a composite end point including also an assessment of nausea, in the aprepitant arm (85% vs 73%; *P* = .06). It is notable that significantly fewer patients (74%) receiving palonosetron alone were free from nausea on day 1. Since the number of nausea‐free patients decreased from day 2 onwards in both treatment arms, it possible that the efficacy of palonosetron may be lost to some extent with additional doses of chemotherapy.^9^ In another study, two extended schedules of palonosetron administered for 5 days (daily or every‐other‐day) were randomly compared with daily ondansetron in 143 patients with AML treated with chemotherapy containing cytarabine at a daily dose of ≥4000 mg/m^2^, which can be considered as highly emetogenic.^9^ Although more patients in the palonosetron arms than in the ondansetron arm achieved a CR (31%, 35%, and 21%, respectively) during the 7‐day study period, the differences were not statistically significant. These findings suggest that palonosetron may not only allow simplification of antiemetic prophylaxis but may also be a more effective 5‐HT_3_RA in this setting. However, if CINV prevention in the setting of chemotherapy for AML must be improved, there are needs to develop multi‐agent regimens that will also improve nausea control. It is encouraging that significantly more patients in the palonosetron plus aprepitant arm reported a CC of nausea than patients in the palonosetron alone arm (43% vs 27%) during the whole study period. In an observational study of 77 patients receiving chemotherapy for AML, when the subgroup of emesis‐free patients was analyzed, even mild nausea was found to be associated with a deterioration of quality of life in 25% of subjects.^6^ In a small study, the benefit of aprepitant has been reported to be more prominent in patients treated with corticosteroid‐containing regimens for hematologic malignancies,^16^ but the overall evidence from our study does not seem to support the view that AML patients treated with mostly corticosteroid‐free regimens derive little benefit from aprepitant. In an attempt to improve the antiemetic prophylaxis, the rate of CR was randomly compared between ondansetron plus aprepitant versus ondansetron alone in 98 AML patients receiving chemotherapy containing cytarabine at a daily dose of ≥1000 mg/m^2^.^17^ In this open‐label study, aprepitant was administered for 4 days, day 1 to 1 day after the end of chemotherapy, and patients were followed for a total of 6 days. Although the rates of CR were similar between the treatment arms, there was only an insignificant increase in the number of aprepitant‐treated patients who were free from nausea throughout the observation study period.

Finally, we performed a complementary analysis using a more rigorous approach assessing preservation of efficacy as it evaluates continued antiemetic protection over the study period by censoring those patients who had a CINV event (ie, treatment failures). This unplanned analysis demonstrated that the time to treatment failure is similar between the two arms in the first 24 hours after chemotherapy initiation. However, in the subsequent days the two curves separate, with significantly more aprepitant‐treated patients having a total control of CINV (ie, no emesis, no use of rescue medication, and no nausea) during the whole study period (log‐rank test; *P* = .03). Since the combination regimen resulted in only 42% of our patients being symptom‐free without use of rescue medications, this analysis also shows that the study population was exposed to a substantial risk of developing CINV during the entire observation period. In a recent single‐arm study, a higher incidence of emesis on the day following completion of the aprepitant was reported in patients treated with 3 + 7 induction chemotherapy for AML and receiving aprepitant for 5 days.^15^ In light of this, the investigators suggested that the administration of aprepitant should be extended beyond day 5 to extend its antiemetic effect. In our study, the significant increase in the proportion of aprepitant‐treated patients with a total control of CINV supports the hypothesis that aprepitant administered for a longer time period could result in better control of symptoms in this challenging setting of CINV.

In conclusion, this study suggests that 3‐day aprepitant added to a simplified regimen of every‐other‐day palonosetron can achieve valuable clinical benefit for the control of CINV in AML patients treated with common induction regimens. However, these results as well as previously reported findings 9 should be considered only as supporting the need for further investigations including every‐other‐day palonosetron in combination with aprepitant given for a longer time period (perhaps for the entire duration of chemotherapy administration) or novel long‐lasting NK‐1RAs to improve CINV control in this setting of multiple‐day, corticosteroid‐free chemotherapy.^18^, ^19^ Further prophylactic measures, such as the use of concomitant olanzapine, should also be evaluated.^20^

## CONFLICT OF INTEREST

Nicola Di Renzo, Lorella Melillo, Fernando Porretto, Michela Dargenio, Vincenzo Pavone, Domenico Pastore, Patrizio Mazza, Donato Mannina, Anxur Merenda, Nicola Cascavilla, Giuseppina Greco, Rosella Matera and Maurizio Musso—none. Erminio Bonizzoni has received consulting fees from Italfarmaco S.p.A. Luigi Celio has received advisory board honoraria from Italfarmaco SpA.

## AUTHOR CONTRIBUTIONS

Nicola Di Renzo: conceptualization, investigation, project administration, resources, supervision, writing of review and editing. Nicola Cascavilla, Vincenzo Pavone, and Maurizio Musso: resources, investigation, writing‐review and editing. Lorella Melillo, Fernando Porretto, Michela Dargenio, Domenico Pastore, Patrizio Mazza, Donato Mannina, Anxur Merenda, Giuseppina Greco and Rosella Matera: resources, writing of review and editing. Luigi Celio: visualization, writing original draft. Erminio Bonizzoni: formal analysis. All authors proofread the article and agreed on the data presented.

## Data Availability

Data are available on request from the authors.
